# Drones in healthcare logistics: Insights from healthcare professionals’ perspective on Zipline delivery services in Ghana

**DOI:** 10.1016/j.dialog.2026.100285

**Published:** 2026-02-02

**Authors:** Emmanuel Komla Dzisi, Morrice Kobby Patterson, Seidu Iddrisu

**Affiliations:** a Department of Civil Engineering, Kwame Nkrumah University of Science and Technology, Kumasi, Ghana; b Regional Transportation Research and Education Center KNUST (TRECK), Kumasi, Ghana

**Keywords:** Drone delivery, Healthcare logistics, Medical supply chain, Zipline Ghana, Health system innovation

## Abstract

The use of drones for healthcare delivery has emerged as a promising innovation for improving access to essential medical supplies, particularly in hard-to-reach areas and during emergencies. This study explores healthcare professionals' perspectives on Zipline's drone services in enhancing healthcare delivery in Ghana, focusing on their awareness, perceived benefits, and challenges. Data was collected through a cross-sectional survey of 696 healthcare professionals across Zipline Ghana's six distribution centers and analyzed using descriptive statistics, chi-square tests of association, and the Kruskal-Wallis test. The results indicate relatively high awareness (62%) of drone services, although facility-level usage was uneven. The findings further reveal that, while drones provide valuable logistical support, they are complementary tools rather than substitutes for broader systemic reforms. Major challenges identified included high operational costs, technical limitations, and concerns about their long-term sustainability. As one of the first large-scale empirical assessments of healthcare professionals' perceptions of drone-enabled healthcare logistics in Sub-Saharan Africa, these findings offer new evidence and policy-relevant insights for Ghana's healthcare system and contribute to broader global discussions on the integration of drones into healthcare delivery.

## 1 Introduction

Globally, access to timely, quality healthcare is a top priority, which is inextricably linked to universal health priorities such as the United Nations Sustainable Development Goals (SDGs). Disparities in healthcare access, which persist especially in regions with infrastructural, logistical, and workforce constraints, were intensified during the COVID-19 pandemic, leaving millions marginalized. The pandemic also exposed the vulnerability of traditional supply chains, underscoring the urgent need for resilient, technology-driven healthcare delivery systems [1], [2], [3].

In Sub-Saharan Africa, health system inefficiencies are worsened by limited health infrastructure, a low doctor-to-population ratio (below the World Health Organization threshold), and geographic inaccessibility of health facilities [4], [5], [6]. Rural facilities often experience stock-outs of basic medicines, vaccines, and emergency supplies [7], [8], undermining progress toward universal health coverage and achievement of SDG Target 3.8, which calls for universal access to essential medicines and health services. To address these barriers, attention has turned to the use of innovative technologies, particularly the deployment of Unmanned Aerial Vehicles (UAVs) to bridge gaps in healthcare logistics [9], [10], [11], [12]. In Rwanda, drones have revolutionized blood delivery, reducing the median delivery time to 41 min. This advancement has resulted in a 67% reduction in blood unit expiration and improved availability in rural hospitals [13], [14]. Comparable initiatives are evident in West Africa and East African countries, where drone technology is being tested to tackle delays in emergency obstetric care, enhance maternal health, and support epidemic response efforts [10], [11], [15].

Despite the growing scholarly work which has made evident the operational importance of medical drones, there is still limited understanding of how healthcare workers in low – and middle-income countries (LMICs) (who are essential for successful implementation), integrate this technology into their daily routine. This study therefore, explores healthcare professionals' perspectives on the role of Zipline's drone services in enhancing healthcare delivery in Ghana. Specifically, it examines healthcare professionals' awareness and adoption of the technology, the perceived benefits for emergency response and medical supply delivery, as well as the challenges faced in drone integration into the current healthcare system. By situating these issues within the broader context of the Sustainable Development Goals (SDGs) and Ghana's National Health Strategies [16], the study aims to close the knowledge gap through empirical, evidence-based, actionable insights for healthcare stakeholders –with implications for sustainable healthcare delivery in Ghana and Sub-Saharan Africa at large.

From an observer's standpoint, Ghana's experience with Zipline's drone delivery system has demonstrated measurable logistical gains, including quicker delivery and improved supply reliability in emergencies. In the Ashanti Region, for instance, facilities utilizing Zipline's services reported a 56.4% reduction in maternal deaths compared to those without drone support [10], [17]. Similarly, rural and hard-to-reach areas have reportedly experienced improvements in efficiency, cost-effectiveness, and accessibility of medical supplies [12], [18]. Atiga et al. [9] also found that the technology significantly reduced delivery lead times and enhanced emergency health services. Yet, the government of the country in December 2025 was reportedly considering the termination of its contract with Zipline, as it claimed the services were not providing enough value for money, carrying other items such as school supplies, books, and non-healthcare-related items, during its operational times. According to the Minister of Health, this had resulted in a total debt of about GH₵175 million (∼$16 million) to the company [19], raising questions about the program's overall usefulness to the state and healthcare in particular. This study, in the attempt to ascertain answers to these questions, sought to assess from the perspective of healthcare workers, their opinions and attitudes about the drone services, their usefulness to their daily operations, and their familiarity with the service in general.

While numerous studies, both in Ghana and beyond show evidence of health workers perceiving medical drones as a practical solution to last-mile bottlenecks in healthcare delivery, especially where distance and supply unreliability hinder service provision [3], [20], [21], [22], [23] few have examined closely, the acceptance and overall effectiveness of such programs. Successfully integrating these services into the national healthcare strategy remains complex and relies on healthcare professionals' awareness, acceptance, and perceptions of the technology. This study aims to address these issues, with the goal of identifying implementation gaps and strengthening the deployment of the service in Ghana, and other similar settings.

### 1.1 Theoretical framework guiding the study

Originally propounded by Davis [24], the Technology Acceptance Model (TAM) explains how individuals form attitudes and intentions toward adopting new technologies. The model proposes that two main factors, which are perceived usefulness (PU) and perceived ease of use (PEOU), influence users' acceptance and actual use of a technology. While perceived usefulness (PU) denotes the extent to which a person believes that a technology enhances job performance, perceived ease of use (PEOU), on the other hand, refers to the degree to which the technology is as effortless to use or integrate into existing work systems [25], [26]. Studies have shown that TAM has been widely applied across education, business, and healthcare research to explain users' behavioral intentions toward technological innovation [27], [28]. Within health and technology research, the model has been used to specifically understand workers acceptance of telemedicine, digital record systems, and mobile health platforms, among others [29], [30], [31], [32]. Consistently, these studies have shown that successful adoption depends not only on the perceived benefits of a technology but also on the institutional and operational conditions that make its use practical and sustainable.

While this current study does not explicitly use TAM, it applies the framework as a conceptual lens to interpret healthcare professionals' awareness, perceived benefits, and challenges of integration of Zipline's drone-enabled medical delivery services in Ghana. In this context, perceived usefulness (PU) pertains to professionals' recognition of drones' ability to improve the timeliness, reliability, and efficiency of medical supply delivery. Conversely, perceived ease of use (PEOU) examines how easily such technology can be integrated into existing healthcare systems while considering factors such as operational costs, regulatory environment, and adequacy of staff training. Positioning the study within this broader TAM framework provides a logical theoretical basis for exploring how perceived benefits and institutional barriers can influence the acceptance and continued use of drone technology in healthcare delivery in Ghana.

## 2 Materials and methods

### 2.1 Study area

**Fig. 1:**
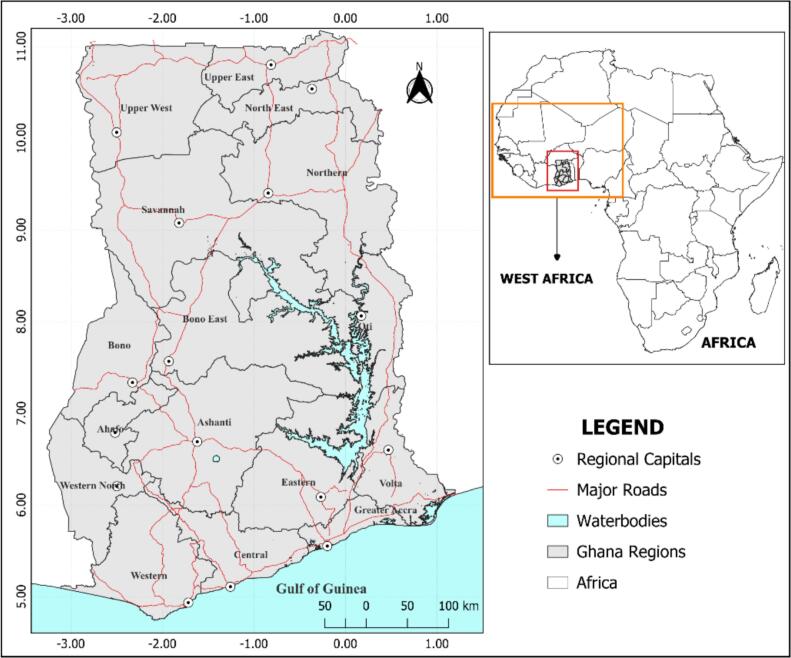
Study area map showing major road networks and regional capitals in Ghana.

Ghana, shown in Fig. 1, is a developing country situated in the West African sub-region of Sub-Saharan Africa with 16 administrative regions: Ashanti, Bono, Bono East, Ahafo, Central, Eastern, Greater Accra, Northern, Savannah, North East, Upper East, Upper West, Volta, Oti, Western, and Western North. The country covers an area of approximately 238,533 km^2^ and shares borders with Burkina Faso to the north, Togo to the east, the Ivory Coast to the west, and the Gulf of Guinea to the south. According to the 2021 Population and Housing Census (PHC) conducted by the Ghana Statistical Services (GSS), Ghana has an estimated population of 31 million, comprising 50.7% females and 49.3% males. The Greater Accra, Ashanti, and Western regions alone, currently account for more than 50% of the entire population, with the country's population projected to reach approximately 35 million by the end of 2025.

Within the West African sub-region, Ghana has the highest healthcare priority index [33], and a fairly balanced distribution of healthcare facilities across its territory, including regional and teaching hospitals, maternity homes, clinics, and Community-based Health Planning and Services (CHPS) compounds that support primary, secondary, and tertiary healthcare services. However, despite this distribution, challenges remain in providing timely and equitable access to care. Factors such as geographic barriers, transportation issues, uneven resource allocation, and a low physician-to-patient ratio continue to hamper healthcare delivery, especially in remote and underserved areas [5], [6], [34].

### 2.2 Zipline distribution centers in Ghana

Zipline's drone-enabled healthcare delivery services began operating in Ghana on April 24, 2019, starting with four distribution centers that later expanded to six. The initiative, called “Fly-to-save-a-life” by the Ministry of Health (MoH), Ghana, aims to create a 24-h medical supply chain using drones, especially for rural and remote areas. Run by Zipline International, the service enables rapid delivery of essential medical supplies such as blood products, vaccines, and other critical items.

**Fig. 2:**
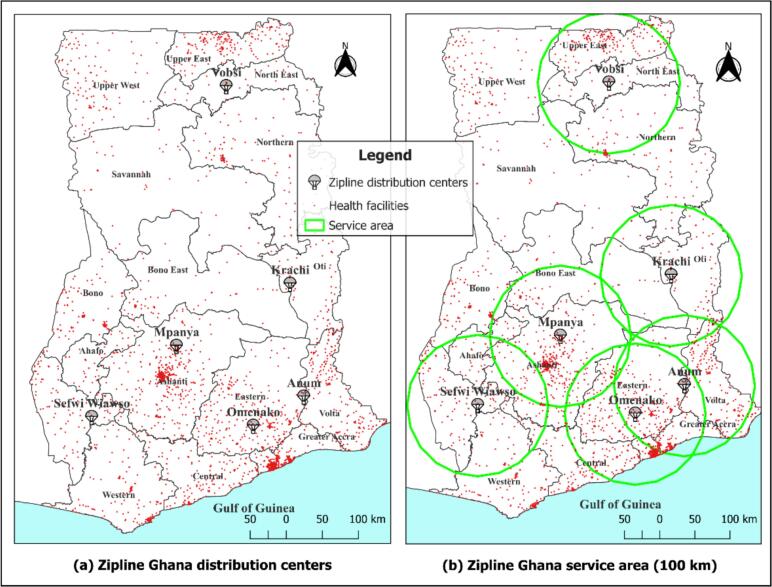
Map showing Zipline Ghana's distribution centers and service areas.

Currently, the six distribution centers operate across the country, collectively covering 13 of Ghana's 16 administrative regions - with only the Savannah, Upper West and Bono regions remaining out of the service's coverage. Each distribution center has a service radius of approximately a 100 km as seen in Fig. 2. These centers are located at Anum, Krachi, Omenako, Sefwi Wiawso, Mpanya, and Vobsi.

The information in Table 1 presents the six (6) Zipline distribution centers in Ghana and the regions they serve.

**Table 1:** Zipline Ghana distribution centers and the Regions they serve.

Distribution centers	Regions served within a 100 km radius
Anum	Eastern, Volta, and Greater Accra
Krachi	Oti and Bono East
Mpanya	Ashanti, Bono East, and Ahafo
Omenako	Eastern, Greater Accra, Central, and Ashanti
Sefwi Wiawso	Western North, Western, Ahafo, and Ashanti
Vobsi	Northern, North East, and Upper East

### 2.3 Population density

**Fig. 3:**
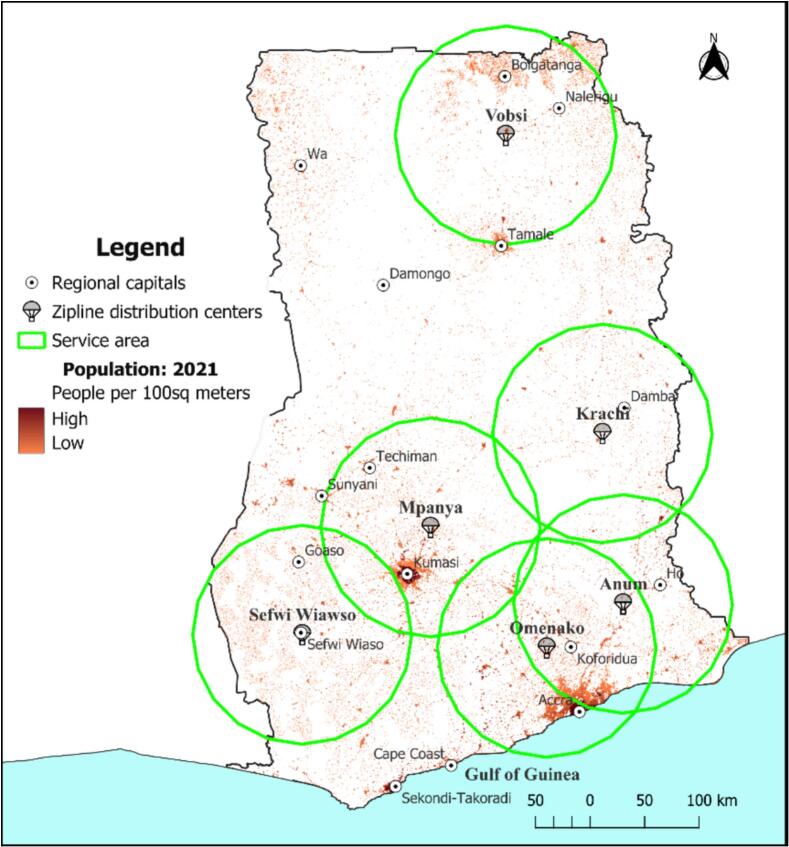
Map showing population densities across Ghana.

Population density across Ghana also varies considerably (as shown in Fig. 3). This uneven population distribution can influence the delivery of essential services like public healthcare delivery services, among others. Using WorldPop gridded population estimates at 100 m spatial resolution, density patterns show very high concentrations in major cities such as Accra, Kumasi, Sekondi–Takoradi, and Tamale, reflecting patterns of urbanization and economic activity. Conversely, the northern and central belts of the country are characterized by low population densities, with widely dispersed settlements.

The six (6) Zipline distribution centers are strategically located to serve both high-density urban areas and low-density rural zones. This positioning is critical for healthcare logistics, as densely populated areas require rapid replenishment of medical supplies to meet high demand, while sparsely populated, remote areas depend on the speed and reach of drone deliveries to overcome transport delays in emergencies and infrastructural issues. The present positioning of centers, while not evenly distributed spatially, still ensures reasonable coverage, timeliness, and equity in access to healthcare resources across most parts of the country.

### 2.4 Study approach

Although the questionnaire contained a small number of open-ended prompts, no formal qualitative analysis (e.g., thematic coding) was undertaken for this paper. The study therefore adopts a purely quantitative analytical framework within a cross-sectional approach. The quantitative design was appropriate because it allowed for the systematic measurement and statistical analysis of healthcare professionals' perspectives on Zipline's drone-enabled medical delivery system. The cross-sectional approach involved collecting data at a single point in time from a defined population, enabling the assessment of existing conditions, patterns, and associations without the need for longitudinal tracking [35]. The design approach was particularly suited for reliably (and simultaneously) carrying out evaluations of service accessibility and operational effectiveness across multiple geographic areas.

### 2.5 Data source

The study employed data from both primary and secondary sources. Primary data were collected from healthcare professionals working in selected health facilities located within the service radii of each Zipline distribution center (see Fig. 2). Secondary data were also obtained from the archives of the Ministry of Health (MoH), Ghana, and included data on all health facilities in Ghana, their geographic locations, and operational details of Zipline's services. Additional population density data at a 100 m spatial resolution were obtained from WorldPop (https://www.worldpop.org/).

### 2.6 Primary data collection technique

Primary data were collected using a semi-structured questionnaire guide administered through KoboCollect; a free, open-source KoboToolbox app designed for data collection. The use of KoboCollect ensured efficient electronic data capture, minimized manual entry errors, and enabled the real-time tracking of enumerators' field activities. The survey instrument was developed through a combination of adapted items from existing literature on healthcare logistics innovation and technology adoption (e.g., [3], [10]), and context-specific items developed by the research team to reflect Ghana's drone-enabled medical delivery system [36]. Items assessing reliance on drone services were informed conceptually by the Technology Acceptance Model, particularly perceived usefulness. Further details on the questionnaire structure and the measurement rationale underpinning the key variables are provided in Section 2.7. Before full deployment, the questionnaire was pretested by the principal investigator and trained enumerators in a small number of health facilities. This pretest was necessary to verify question clarity, confirm cultural appropriateness, estimate the time needed for completion, and identify any technical issues with the data collection tool. Each questionnaire took about 40 min to complete. The entire data collection process spanned twelve weeks, ensuring sufficient time to reach facilities across the geographic zones covered by the distribution centers. Ethical integrity was maintained throughout the study. Participation was voluntary, and informed consent was obtained from all respondents before data collection. Respondents were assured of the confidentiality of their information with no identifying details in the dataset. Ethical review of the survey instrument was provided by the Committee on Human Research, Publication and Ethics (CHRPE) at the Kwame Nkrumah University of Science and Technology (KNUST), Ghana, under reference number CHRPE/AP/1101/25. Data for the study were stored electronically and used solely for the purposes of this research.

### 2.7 Questionnaire design and measurement justification

The questionnaire consisted of structured items capturing healthcare professionals' awareness, perceived usefulness, and reported reliance on drone delivery services across distinct healthcare service domains. Items assessing reliance were intentionally designed as stand-alone indicators, each corresponding to a specific operational function of drone services (e.g., routine medical supply delivery, emergency response, laboratory sample transport, and blood delivery), rather than as reflective indicators of a single latent construct. Because these items represent functionally distinct service domains, internal consistency reliability measures such as Cronbach's alpha were not considered appropriate, as such statistics assume undimensionality and interchangeable indicators. Instead, measurement quality was ensured through domain-based item construction, expert review for content validity, and pretesting to confirm clarity and relevance of each item within its respective operational context.

Content validity was assessed through expert review involving transport and public health researchers with experience in drone-enabled healthcare logistics, who evaluated item relevance, wording, and domain alignment. Pretesting with a subset of healthcare professionals informed minor refinements to item phrasing and response options. Additionally, low item non-response rates across the reliance questions provided indirect evidence of item clarity and respondent comprehension.

### 2.8 Sample size and sampling technique

A total of 696 healthcare professionals participated in the study and these healthcare workers were sampled using a stratified sampling technique. The stratified sampling approach was adopted to ensure adequate representation from health facilities within the service coverage of each Zipline distribution center. The approach also ensured a balanced representation of the sample, reflecting the geographic and operational diversity of Zipline's service areas, ultimately, improving the external validity of the results. The unit of analysis for the study is the individual healthcare professional respondent. Although multiple respondents may have been drawn from the same health facility, the analysis focuses on individual perceptions rather than facility-level outcomes. The breakdown of respondents by service area was as follows: Anum (*n* = 105), Vobsi (*n* = 75), Mpanya (*n* = 146), Omenako (*n* = 224), Sefwi Wiawso (*n* = 87), and Krachi (*n* = 59).

### 2.9 Data analysis techniques

Data collected were cleaned and organized in Microsoft Excel and analyzed using Statistical Package for the Social Sciences (SPSS) version 27. Descriptive statistics, including frequencies and percentages, were used to summarize the respondents' socio-economic and demographic characteristics, as well as key categorical variables.

For variables such as awareness of the drone program at health facilities, staff perceptions of benefits of the drone program, and challenges of the program, responses were recorded as binary outcomes (“No” or “Yes”). Chi-square tests of association were applied to these categorical variables to assess whether there were statistically significant relationships between facility location and the various perceptions/experiences of healthcare professionals. The Chi-square tests of association were considered suitable given the nominal nature of these variables and the study's aim to explore associations rather than to predict causality. On the other hand, the Kruskal-Wallis test was employed when intending to assess healthcare professionals' reliance on drone programs for healthcare support, with respondents rating their agreement with several statements on a four-point Likert scale from not reliant (1) to reliant (4). The Kruskal–Wallis test, which is a non-parametric test, was used because it does not assume interval-level measurement or normality. Median scores, Interquartile Range (IQRs), H-statistic, *p*-value, and effect size (**ε**^**2**^**)** were reported in conjunction with this test. The study, therefore in doing this, adopted a purely quantitative analytical framework. Statistical significance was assessed at the 5% level (α = 0.05). The Likert-scale items assessing reliance on drone services were analyzed as separate functional domains. Further justification for this measurement approach is provided in Section 2.7.

## 3 Results and discussion

### 3.1 Background characteristics of respondents and health facilities information

Table 2 presents the summary demographic statistics of healthcare professionals and health facilities sampled within the service radii of the six Zipline Ghana distribution centers. The majority of respondents were female (58.5%), consistent with gendered patterns in Ghana's health workforce, with the rest self-identifying as male. This trend was fairly uniform across sampled health facilities within the distribution centers' service radii, though the gender gap was most pronounced in Krachi, which had 67.8% females, and least in Omenako with 54% females. With respect to professional roles, nurses formed the largest group (39.1%), followed by midwives (13.5%) and administrative and support staff (15.5%). Doctors represented only a small fraction of the sample (1.7%). The high share of nurses was consistent across centers. Mpanya recorded the highest proportion of administrative staff (25.3%) compared to other locations, while Krachi (18.6%) and Sefwi Wiawso (17.2%) had higher proportions of midwives, and Anum (9.5%) had the highest concentration of pharmacists.

**Table 2:** Summary statistics of respondents and health facilities characteristics.

Variable/Category	Combined	Omenako	Anum	Mpanya	Sefwi Wiawso	Vobsi	Krachi
	N (%)	N (%)	N (%)	N (%)	N (%)	N (%)	N (%)
**Gender of the Respondent**							
Male	289(41.5)	103(46)	43(41)	59(40.4)	36(41.4)	29(38.7)	19(32.2)
Female	407(58.5)	121(54)	62(59)	87(59.6)	51(58.6)	46(61.3)	40(67.8)
**The role of the Respondent in the Hospital**							
Doctor	12(1.7)	5(2.2)	1(1)	2(1.4)	1(1.1)	2(2.7)	1(1.7)
Nurse	272(39.1)	83(37.1)	42(40)	57(39)	36(41.4)	32(42.7)	22(37.3)
Midwife	94(13.5)	29(12.9)	14(13.3)	14(9.6)	15(17.2)	11(14.7)	11(18.6)
Pharmacist	43(6.2)	12(5.4)	10(9.5)	8(5.5)	6(6.9)	4(5.3)	3(5.1)
Allied Health Professional	53(7.6)	18(8)	8(7.6)	12(8.2)	5(5.7)	6(8)	4(6.8)
Administrative and Support Staff	108(15.5)	35(15.6)	14(13.3)	37(25.3)	8(9.2)	6(8)	8(13.6)
Public Health Worker	44(6.3)	15(6.7)	5(4.8)	7(4.8)	5(5.7)	6(8)	6(10.2)
Technical and Engineering Staff	38(5.5)	15(6.7)	7(6.7)	4(2.7)	6(6.9)	4(5.3)	2(3.4)
Social Worker/Counsellor	30(4.3)	10(4.5)	4(3.8)	5(3.4)	5(5.7)	4(5.3)	2(3.4)
Physician Assistant	2(0.3)	2(0.9)	–	–	–	–	–
**Number of Years worked in the health sector**							
Less than 3 years	350(50.3)	117(52.2)	47(44.8)	65(44.5)	48(55.2)	41(54.7)	32(54.2)
4–6 years	246(35.3)	75(33.5)	42(40)	57(39)	29(33.3)	23(30.7)	20(33.9)
7–10 years	39(5.6)	13(5.8)	5(4.8)	11(7.5)	5(5.7)	3(4.0)	2(3.4)
Above 10 years	61(8.8)	19(8.5)	11(10.5)	13(8.9)	5(5.7)	8(10.7)	5(8.5)
**Ownership situation of the healthcare centre**							
Privately Owned	46(12.9)	18(14.9)	4(8)	16(21.1)	4(9.8)	2(5)	2(7.1)
Government-Owned	278(78.1)	95(78.5)	41(82)	53(69.7)	32(78)	34(85)	23(82.1)
Partnership	32(9)	8(6.6)	5(10)	7(9.2)	5(12.2)	4(10)	3(10.7)
**Health facility's staff strength**							
Less than 150 staff	146(41)	51(42.1)	19(38)	32(42.1)	16(39)	18(45)	10(35.7)
150–250 staff	86(24.2)	29(24)	14(28)	16(21.1)	10(24.4)	11(27.5)	6(21.4)
251–350 staff	60(16.9)	21(17.4)	7(14)	13(17.1)	8(19.5)	5(12.5)	6(21.4)
351–450 staff	24(6.7)	8(6.6)	2(4)	6(7.9)	4(9.8)	2(5)	2(7.1)
451–550 staff	12(3.4)	2(1.7)	4(8)	2(2.6)	1(2.4)	1(2.5)	2(7.1)
Above 550 staff	28(7.9)	10(8.3)	4(8)	7(9.2)	2(4.9)	3(7.5)	2(7.1)

Footnote: Percentages are based on the number of valid responses for each item; totals may not sum to the overall sample size (N = 696) due to item non-response.

About half of the respondents (50.3%) had worked in the health sector for less than three years, while just over one-third (35.3%) had four to six years of experience. Only 8.8% had more than ten years of service. This indicates a predominantly young workforce, a pattern consistent across all distribution-center service radii. The majority of respondents worked in government-owned health facilities (78.1%), followed by those in privately owned facilities (12.9%) and those in partnership facilities (9%). Vobsi and Krachi had the highest concentration of government facilities (85% and 82.1%, respectively), while Mpanya recorded, proportionally, the highest share of private facilities (21.1%). Regarding staff capacity, most facilities (41%) had fewer than 150 staff, followed by those with 150–250 staff (24.2%). Only 7.9% of facilities reported more than 550 staff. Distribution center-specific differences were modest, though Krachi had a slightly higher concentration (21.4%) of medium-sized facilities (251–350 staff) compared to others.

### 3.2 Awareness and adoption of Zipline Ghana drone delivery services

The findings in Table 3 demonstrate moderately high awareness of drone-enabled healthcare delivery, with 62.2% of healthcare professionals across sampled facilities indicating that they are aware of Zipline's program. However, a considerable share (37.8%) reported no awareness, suggesting uneven dissemination of information to frontline staff. Awareness was relatively consistent across the distribution centers, ranging from 55.2% in the Sefwi Wiawso area to 67.1% in Mpanya; however, these differences were not statistically significant at the 0.05 level (X^2^ = 5.331, *p* = 0.377).

**Table 3:** Awareness of drone programs and service improvements at health facilities.

		Distribution centers						Chi-square test	
Variable/category	Combined	Omenako	Anum	Mpanya	Sefwi Wiawso	Vobsi	Krachi	X^2^	P-value
	N (%)	N (%)	N (%)	N (%)	N (%)	N (%)	N (%)		
**Awareness of Drone-Based Healthcare Delivery**									
No	263(37.8)	81(36.2)	46(43.8)	48(32.9)	39(44.8)	28(37.3)	21(35.6)	5.331	0.377
Yes	433(62.2)	143(63.8)	59(56.2)	98(67.1)	48(55.2)	47(62.7)	38(64.4)		
**Health facility drone utilization**									
No	231(53.3)	80(55.9)	27(45.8)	65(66.3)	19(39.6)	19(40.4)	21(55.3)	17.566	0.063
Yes	157(36.3)	49(34.3)	27(45.8)	26(26.5)	23(47.9)	20(42.6)	12(31.6)		
Unaware	45(10.4)	14(9.8)	5(8.5)	7(7.1)	6(12.5)	8(17)	5(13.2)		
**Patients served (monthly)**									
Less than 5 patients	19(16.5)	6(16.7)	4(21.1)	4(22.2)	3(17.6)	2(11.8)	0(0)	5.444	0.988
5–20 patients	49(42.6)	15(41.7)	7(36.8)	7(38.9)	8(47.1)	9(52.9)	3(37.5)		
21–50 patients	25(21.8)	8(22.2)	5(26.3)	4(22.2)	3(17.6)	2(11.8)	3(37.5)		
More than 50 patients	22(19.1)	7(19.4)	3(15.8)	3(16.7)	3(17.6)	4(23.5)	2(25)		
**Improvements since the inception/adoption of the drone for healthcare delivery program**									
**Medical Supply Delivery**									
No	72(45.9)	21(42.9)	13(48.1)	13(50)	12(52.2)	9(45)	4(33.3)	1.548	0.907
Yes	85(54.1)	28(57.1)	14(51.9)	13(50)	11(47.8)	11(55)	8(66.7)		
**Emergency Response**									
No	78(49.7)	22(44.9)	14(51.9)	15(57.7)	13(56.5)	8(40)	6(50)	2.348	0.799
Yes	79(50.3)	27(55.1)	13(48.1)	11(42.3)	10(43.5)	12(60)	6(50)		
**Routine Health Delivery Services**									
No	111(70.7)	34(69.4)	19(70.4)	22(84.6)	15(65.2)	13(65)	8(66.7)	3.214	0.667
Yes	46(29.3)	15(30.6)	8(29.6)	4(15.4)	8(34.8)	7(35)	4(33.3)		
**Telemedicine**									
No	137(87.3)	43(87.8)	22(81.5)	23(88.5)	21(91.3)	18(90)	10(83.3)	1.496	0.914
Yes	20(12.7)	6(12.2)	5(18.5)	3(11.5)	2(8.7)	2(10)	2(16.7)		

Footnote: Questions about observed improvements were answered only by healthcare facilities which had adopted the service (i.e. N = 157).

In terms of adoption, only 36.3% of respondents reported that their health facilities used drones, while 53.3% indicated non-use and 10.4% were unaware of their facility's usage status. Utilization was highest in Sefwi Wiawso (47.9%) and Anum (45.8%), but lowest in Mpanya (26.5%). Although the chi-square test did not confirm a statistically significant association (X^2^ = 17.566, *p* = 0.063), the patterns point to facility-level and institutional variations rather than simple geographic differences.

According to Kremer et al. [10] and Ssemata et al. [11], in some cases, while drone logistics systems are visible at the national level, gaps in sensitization at the facility level remain a challenge, which can impact overall project efficiency. Based on the strength of the responses from hospital staff (who are key stakeholders for such a program), the authors of this present study see these results are consistent with the hypothesis of earlier authors (Kremer et al. [10]; Ssemata et al. [11]). Furthermore, the findings of Agyei & Kumah [7] and Kremer et al. [8] which suggested that the integration of drones into health systems requires more than technical availability but also facility readiness, system adaptation, and supportive administrative structures appear to be true as the existence of the drone service in this study alone does not seem to have translated into awareness or full adoption of the service.

Among the facilities using the service, patient reach remained modest. The majority (42.6%) reported serving between 5 and 10 patients per month, while 21.8% served 21 and 50, and 19.1% served more than 50 patients a month using this service. Only 16.5% served fewer than five patients. The similarity across centers, with no significant association (X^2^ = 5.444, *p* = 0.988), suggests that even in facilities adopting drones, integration into routine healthcare delivery is still limited in scale. This reflects early adoption dynamics, where technology is often used for specific, high-priority needs rather than broad, systemic operations [1], [9]. Perception of service improvement since adoption is mixed. A slight majority perceived improvements in medical supply delivery (54.1%) and emergency response (50.3%), highlighting drones' strengths in bridging the last-mile supply gap [9]. However, fewer respondents observed improvements in routine service delivery (29.3%) and telemedicine (12.7%), suggesting that the benefits of drones remain concentrated in emergency logistics rather than transforming broader clinical or telehealth systems. Importantly, no significant differences were observed across centers, reinforcing that these perceptions were systemic rather than localized. The findings mirror evidence from Rwanda and Uganda, where drones have been most impactful in reducing stock-outs and emergency delays but less integrated into routine or telemedicine services [11], [14].

Collectively, these results point to a gap between awareness and adoption. While the majority of healthcare professionals are aware of drone services, fewer than four in ten report facility-level utilization. Moreover, patient reach and perceived improvements indicate that integration remains limited in scope, as noted in recent studies and reports in Ghana [19]. The high level of awareness observed among healthcare professionals may however, likely reflect Zipline's nationwide presence and media visibility. Nevertheless, awareness alone does not appear to have translated into widespread adoption. Ghana, as in the case in Rwanda, is experiencing high drone familiarity but has achieved far less integration into mainstream health worker workflows [13], [15]. This suggests that, continued professional sensitization, logistical coordination, and supportive national policy frameworks may be crucial to the full-scale adoption and effective integration/use of drone technology in healthcare delivery.

Table 4, presents the results of the Kruskal-Wallis test with median and Inter-Quartile Range (IQR) scores generated from the 4-point Likert-scale (not reliant to reliant). Reported reliance on drone services is presented separately across service domains, reflecting the questionnaire design in which these items were treated as distinct indicators rather than combined into a composite scale. The results show a moderate level of reliance on drone services for medical supply delivery, routine health delivery services and emergency response. Respondents generally perceived their healthcare facilities as moderately reliant (median = 3) on drones for medical supply delivery, with relatively consistent views (IQR = 1), and no strong polarization toward the extremes across all centers.

**Table 4:** Reliance on drone programs for healthcare support.

	Distribution centers						Kruskal-Wallis Test	
Variable/category	Omenako	Anum	Mpanya	Sefwi Wiawso	Vobsi	Krachi	H-Statistic	P-value Effect size (ε^2^)
	Median (IQR)	Median (IQR)	Median (IQR)	Median (IQR)	Median (IQR)	Median (IQR)		
Medical Supply Delivery	3.0(1.0)	3.0(1.0)	3.0(1.0)	3.0(1.0)	3.0(1.5)	3.0(1.0)	7.82 (df = 5)	0.167 (0.023 (small)
Emergency Response	3.0(2.0)	3.0(2.0)	3.0(2.0)	3.0(2.0)	3.0(2.0)	3.0(2.0)	10.34 (df = 5)	0.066 (0.03 small)
Telemedicine	2.0(2.0)	2.0(2.0)	2.0(2.0)	2.0(2.0)	2.0(2.0)	2.0(2.0)	8.73 (df = 5)	0.120 (0.029 small)
Routine Health Delivery Services	3.0(2.0)	3.0(2.0)	3.0(2.0)	3.0(2.0)	3.0(2.0)	3.0(2.0)	6.54 (df = 5)	0.257 (0.018 small)

Footnote: The Likert-scale items assessing reliance on drone services were analyzed as distinct functional domains (medical supply delivery, emergency response, routine services, and telemedicine) rather than as a single composite scale. As such, internal consistency measures (e.g., Cronbach's alpha) were not computed, since the items were not intended to represent a unidimensional construct.

Respondents also perceived their facilities as being moderately reliant (median = 3) on drone programs for Emergency Response and Routine Health Delivery Services, although there was relatively less consistency observed among these views (IQR = 2). Low reliance (median = 2) was also reported for Telemedicine services, with less consistency among views (IQR = 2).

These results confirm prior findings, which showed drones' impact in reducing medical supply delays and stock-outs in Ghana and Rwanda [10], [13], although slightly lower integration existed for broader health system functions [3], [11], such as routine health service delivery and telemedicine. Importantly, there were no statistically significant differences between distribution centers (*p* > 0.05), suggesting that these perceptions are system-wide rather than location-specific. For emergency response reliance, results suggested a non-significant trend toward differences across distribution centers (*p* = 0.066), but this did not meet conventional thresholds for statistical significance. As such, no post-hoc tests were conducted as *p* > 0.05.

### 3.3 Perceived benefits in emergency response and supply delivery

Table 5 highlights healthcare professionals' recognition of the benefits of the drone program for healthcare delivery, although perceptions vary depending on the specific function.

**Table 5:** Facility staff perceived the benefits of drones for healthcare delivery.

		Distribution centers						Chi-square test	
Variable/category	Combined	Omenako	Anum	Mpanya	Sefwi Wiawso	Vobsi	Krachi	X^2^	P-value
	N (%)	N (%)	N (%)	N (%)	N (%)	N (%)	N (%)		
**Improved the response times in Emergencies**									
No	52(33.1)	18(36.7)	6(22.2)	12(46.2)	6(26.1)	5(25)	5(41.7)	5.235	0.388
Yes	105(66.9)	31(63.3)	21(77.8)	14(53.8)	17(73.9)	15(75)	7(58.3)		
**Reducing supply chain gaps**									
No	105(66.9)	33(67.3)	16(59.3)	17(65.4)	17(73.9)	14(70)	8(66.7)	1.341	0.931
Yes	52(33.1)	16(32.7)	11(40.7)	9(34.6)	6(26.1)	6(30)	4(33.3)		
**Timely Delivery of Medical Supplies**									
No	30(19.1)	10(20.4)	5(18.5)	5(19.2)	4(17.4)	3(15)	3(25)	0.592	0.988
Yes	127(80.9)	39(79.6)	22(81.5)	21(80.8)	19(82.6)	17(85)	9(75)		
**Perceived Improvement in Healthcare Access in Rural Areas**									
No	106(67.5)	34(69.4)	16(59.3)	20(76.9)	15(65.2)	12(60)	9(75)	2.844	0.724
Yes	51(32.5)	15(30.6)	11(40.7)	6(23.1)	8(34.8)	8(40)	3(25)		

Footnote: Questions about observed improvements were answered only by healthcare facilities that had adopted the service (i.e. N = 157).

The majority of respondents (66.9%) reported that the drone program had improved response times in emergencies, underscoring its perceived value in situations where rapid delivery of supplies is critical. This perception was relatively consistent across distribution centers, with the highest positive responses occurring at the health centers near Anum (77.8%), and Sefwi Wiawso (73.9%), while the least positive responses were recorded at Mpanya (53.8%). Differences in perception across centers were not statistically significant (X^2^ = 5.235, *p* = 0.388). Previous studies in Rwanda and Ghana found similar results, where drone technology was particularly well-suited for time-sensitive emergencies such as maternal hemorrhage and blood shortages [10], [14]. Few respondents (33.1%) agreed that drones had been effective in reducing supply chain gaps, with two-thirds (66.9%) indicating otherwise. The limited agreement on this benefit may suggest that while drones fill urgent, last-mile delivery needs, they are not perceived as a comprehensive solution to wider supply chain challenges [9], [11], [18]. This also confirms earlier research findings, which highlight drones' abilities to address emergency shortages, but their limited capacity to resolve longer-term supply chain resilience issues.

The most strongly perceived benefit was seen in the drone program's ability to ensure timely delivery of medical supplies, with 80.9% of respondents affirming this. This perception was strikingly uniform across centers, with positive responses ranging from 75% in Krachi to 85% in Vobsi. The chi-square test confirmed no significant regional variation (X^2^ = 0.592, *p* = 0.988). These findings reinforce the widely documented advantage of drones in overcoming transport delays and geographic barriers to ensure that essential commodities such as vaccines, blood products, and emergency medicines reach facilities promptly [1], [9]. With regard to rural areas in particular, only about one-third of respondents (32.5%) perceived that drones had improved healthcare access in such areas, while the majority (67.5%) did not perceive any significant changes had occurred, even with the emergence of this program. These perceptions were broadly consistent across centers, with slightly more positive responses in Anum (40.7%) and Sefwi Wiawso (34.8%) compared with Mpanya (23.1%). This suggests that while drones may enhance logistics, they are not seen as directly addressing structural barriers to rural health access, such as limited workforce, inadequate infrastructure, or long travel times to facilities [6], [7], [8].

### 3.4 Challenges affecting the integration and use of Zipline Ghana's drone delivery services

The integration of drone technology into Ghana's healthcare delivery system has not been without challenges. These challenges were identified by healthcare professionals across all staff categories, as presented in Table 6. The high initial and operational costs emerged as the most pressing issue, with 68% of respondents identifying this as a significant challenge. This concern was consistently observed across distribution centers, with especially negative outlooks in Mpanya (73%) and Krachi (73.3%). Cost concerns reflect broader regional debates in Sub-Saharan Africa, where drone programs frequently rely on donor or state subsidies for survival [1].

**Table 6:** Perceived challenges in the integration of Zipline drone services.

		Distribution centers						Chi-square test	
Variable/category	Combined	Omenako	Anum	Mpanya	Sefwi Wiawso	Vobsi	Krachi	X^2^	P-value
	N (%)	N (%)	N (%)	N (%)	N (%)	N (%)	N (%)		
**High Initial/Operational Costs**									
No	171(32.0)	50(29.1)	31(38.8)	31(27)	28(43.1)	19(33.3)	12(26.7)	7.995	0.157
Yes	363(68.0)	122(70.9)	49(61.3)	84(73)	37(56.9)	38(66.7)	33(73.3)		
**Technical Limitations e.g., battery life, payload capacity**									
No	210(44.7)	60(40)	32(43.8)	48(51.1)	27(44.3)	19(36.5)	24(60)	8.097	0.151
Yes	260(55.3)	90(60)	41(56.2)	46(48.9)	34(55.7)	33(63.5)	16(40)		
**Maintenance and Operational Challenges**									
No	267(61.2)	80(56.7)	41(62.1)	55(61.1)	37(67.3)	26(56.5)	28(73.7)	4.98	0.418
Yes	169(38.8)	61(43.3)	25(37.9)	35(38.9)	18(32.7)	20(43.5)	10(26.3)		
**Security Concerns**									
No	276(59.0)	83(56.1)	42(57.5)	60(65.9)	37(59.7)	27(51.9)	27(64.3)	3.967	0.554
Yes	192(41.0)	65(43.9)	31(42.5)	31(34.1)	25(40.3)	25(48.1)	15(35.7)		
**Lack of Public and Stakeholder Acceptance**									
No	249(60.0)	71(53.4)	42(63.6)	53(60.9)	34(66.7)	23(53.5)	26(72.2)	6.754	0.24
Yes	167(40.0)	62(46.6)	24(36.4)	34(39.1)	17(33.3)	20(46.5)	10(27.8)		
**Environmental Factors**									
No	259(55.8)	76(52.4)	40(54.1)	58(63.7)	34(55.7)	23(45.1)	28(66.7)	7.469	0.188
Yes	205(44.2)	69(47.6)	34(45.9)	33(36.3)	27(44.3)	28(54.9)	14(33.3)		
**Regulatory Hurdles**									
No	278(64.4)	81(60)	44(64.7)	61(67.8)	37(67.3)	28(60.9)	27(71.1)	2.77	0.735
Yes	154(35.6)	54(40)	24(35.3)	29(32.2)	18(32.7)	18(39.1)	11(28.9)		

Footnote: Percentages are based on the number of valid responses for each item; totals may not sum to the overall sample size (N = 696) due to item non-response.

Technical limitations were also notable. More than half of the respondents (55.3%) cited technical limitations, such as battery life and payload capacity as a constraint, though this was not uniformly observed across centers. For instance, Vobsi reported relatively high concern (63.5%), while Krachi reported a lower level (40%) of concern. Other challenges were mentioned less frequently but still merit attention. About 41% of respondents identified security concerns, while 40% pointed to a lack of public and stakeholder acceptance. Security issues for instance, might not have been perceived as a problem by respondents, perhaps because the drones were not directly the responsibility of the healthcare facilities but rather the drone company Zipline. These perceptions, however, varied modestly across centers but did not show statistically significant differences. Interestingly, regulatory hurdles were reported by just over a third of respondents (35.6%), reflecting persistent uncertainties around airspace regulation and drone governance. The challenges identified show that the success of drone integration depends not only on technological efficiency but also on institutional alignment [11]. These findings suggest that the sustainability of Zipline's drone services hinges less on awareness and more on willingness to adopt, financial viability, technical scalability, and systemic integration. While drones are seen as innovative and useful, their long-term role in Ghana's health system will depend on their operational costs, expanded technical capacity, and the embedding of services within a clear policy and financing framework [12], [37].

### 3.5 Results and discussion summary

This study examined healthcare professionals' perceptions of Zipline's drone-enabled medical delivery system and its integration into Ghana's healthcare delivery framework. The findings reveal a generally high level of awareness and a moderate level of adoption, with drones primarily associated with improvements in emergency response and rapid medical supply delivery. However, their perceived impact on broader supply chain resilience and healthcare access—especially in rural areas—remains limited.

Interpreted through the lens of the Technology Acceptance Model (TAM), these results suggest that healthcare professionals perceive drones as useful for addressing critical delivery gaps but face institutional, logistical, and operational barriers that affect their perceived ease of use [28]. This finding underscores that technological acceptance in healthcare systems is not merely a function of individual attitudes, but of institutional readiness, policy support, and system integration. Facilities with better administrative coordination and resource support are more likely to demonstrate higher adoption and reliance, as technological innovations thrive where organizational infrastructure is adaptive and well-aligned.

From a broader theoretical perspective, these findings reinforce the continued relevance of Rogers' innovation diffusion theory, highlighting how the five steps of innovation, namely—awareness, interest, evaluation, trial and adoption remain essential for acceptance of new innovations. It is worth noting that while these remain relevant, new, similarly developed programs will have a higher chance of success when embedded in social and institutional ecosystems that support their systemic adoption. Ghana's healthcare drone adoption case should perhaps mirror experiences in Rwanda and Uganda, where drone programs achieved stronger integration through centralized coordination, government partnerships, and policy coherence [11], [14].

Recent findings from the Ghanaian Ministry of Health's ongoing governmental review of Zipline Ghana's *Fly-To-Save-A-Life* initiative however suggest this is presently not the case. The review indicates that three distribution centers are at risk of closure, while the service currently reaches only an estimated 12% of designated hard-to-reach communities (even lower than the 36.3% adoption rate reported in this present study). Moreover, emergency medical deliveries reportedly constitute a minority of total deliveries. These figures raise substantive concerns about the depth of the program's integration into the national health system and its overall sustainability. Importantly, these policy-level observations corroborate the perceptions reported by healthcare facilities in this study, which highlighted limited institutional embedding and uneven reliance on the drone service within routine healthcare delivery.

Thus, while Zipline's model demonstrates technological feasibility and operational value, Ghana's experience underscores a crucial lesson: innovation alone does not guarantee transformation. Without systemic integration, sustained funding, and human capital investment, drone technology risks remaining a niche logistical solution rather than a transformative component of the national health system.

## 4 Policy implications

The results highlight that the sustainability of drone-based medical delivery depends on moving from technological novelty to institutional maturity. To ensure long-term impact, Ghana's health policymakers must focus on governance, financing, and human systems alongside technology. Three (3) strategic priorities are proposed:

a) Institutionalization:
Drone logistics should transition from project-based innovation to a core component of the Ministry of Health (MoH) and Ghana Health Service (GHS) frameworks. Embedding drone operations within national programs particularly in emergency response, maternal health, and pharmaceutical supply chains will promote coordination, funding continuity, and national ownership.
b) Regulatory Coherence and Governance:
  Current drone regulations emphasize aviation safety but lack robust provisions for medical logistics, data ethics, and supply chain governance. Establishing a multi-agency framework involving the Ghana Civil Aviation Authority (GCAA), Food and Drugs Authority (FDA), and Ghana Health Service (GHS) can harmonize oversight, strengthen interoperability, and ensure accountability across the value chain.
c) Capacity and Trust Building:
  Effective integration depends on professional confidence and technical competence. Continuous training, participatory planning, and inclusion of drone logistics in medical and public health curricula across the country can enhance health workers' operational literacy and trust for such systems. Additionally, public sensitization campaigns can reduce skepticism and promote acceptance at the community level.

### 4.1 Study limitations

This study has several limitations that should be acknowledged when interpreting the findings. First, the cross-sectional design provides a snapshot of healthcare professionals' perceptions at a single point in time and does not allow for temporal analysis or causal inference regarding changes in awareness, adoption, or reliance on drone delivery services as the program matures.

Second, the analysis relies primarily on self-reported perceptions of healthcare professionals, which may be subject to recall bias or social desirability bias, particularly given the high-profile and government-supported nature of the Zipline program. Therefore, reported perceptions may not fully reflect actual operational reliance or effectiveness.

Third, the study does not incorporate objective performance or outcome indicators—such as delivery times, cost-effectiveness measures, service reliability metrics, or patient health outcomes—which limits the assessment of the measurable impact of drone-enabled delivery beyond perceived benefits.

Fourth, the analytical approach is largely descriptive and bivariate, employing chi-square and non-parametric tests to examine associations across service areas. The absence of multivariate or explanatory modeling limits deeper inference regarding the relative influence of institutional, geographic, or professional factors on adoption and reliance patterns.

Finally, while the focus on healthcare professionals provides critical frontline insights, the exclusion of policymakers, drone operators, and community stakeholders limits a more holistic understanding of system-level integration and sustainability. Future research would benefit from longitudinal or mixed-method designs that combine perception-based data with objective performance metrics and multi-stakeholder perspectives. Comparative studies across other Zipline-operating countries, such as Rwanda and Nigeria, could further elucidate how differing policy, economic, and infrastructural contexts shape scale-up and long-term viability.

## 5 Conclusion

In conclusion, Zipline's drone delivery system is perceived as useful but only partially impactful based on the assessment of healthcare workers in the country. Its transformative potential remains constrained by financial, technical, and institutional factors. The findings emphasize that technological adoption in healthcare is a systemic process, dependent not just on innovation, but on governance, workforce engagement, and sustainable financing.

To translate promise into permanence, Ghana must move beyond technology-centered enthusiasm toward policy-anchored integration. With coherent regulatory frameworks, institutional alignment, and strategic investment in capacity and equity, drones can evolve from a symbolic innovation to a critical infrastructural pillar in Ghana's and more broadly, Africa's healthcare delivery ecosystem.

## CRediT authorship contribution statement

**Emmanuel Komla Dzisi:** Writing – review & editing, Writing – original draft, Visualization, Supervision, Methodology, Formal analysis, Data curation, Conceptualization. **Morrice Kobby Patterson:** Writing – original draft, Visualization, Methodology, Formal analysis, Data curation, Conceptualization. **Seidu Iddrisu:** Writing – review & editing, Writing – original draft, Visualization, Formal analysis.

## Declaration of competing interest

The authors declare that they have no known competing financial interests or personal relationships that could have appeared to influence the work reported in this paper.
